# Chronic impact of traumatic brain injury on outcome and quality of life: a narrative review

**DOI:** 10.1186/s13054-016-1318-1

**Published:** 2016-06-21

**Authors:** Nino Stocchetti, Elisa R. Zanier

**Affiliations:** Department of Physiopathology and Transplantation, Milan University, Milan, Italy; Neuro ICU Fondazione IRCCS Cà Granda Ospedale Maggiore Policlinico, Via F Sforza, 35, 20122 Milan, Italy; Department of Neuroscience, IRCCS—Istituto di Ricerche Farmacologiche Mario Negri, via Giuseppe La Masa 19, 20156 Milan, Italy

**Keywords:** Traumatic brain injury, Long-term outcome, Quality of life, Rehabilitation, Disability, Axonal injury

## Abstract

Traditionally seen as a sudden, brutal event with short-term impairment, traumatic brain injury (TBI) may cause persistent, sometimes life-long, consequences. While mortality after TBI has been reduced, a high proportion of severe TBI survivors require prolonged rehabilitation and may suffer long-term physical, cognitive, and psychological disorders. Additionally, chronic consequences have been identified not only after severe TBI but also in a proportion of cases previously classified as moderate or mild. This burden affects the daily life of survivors and their families; it also has relevant social and economic costs.

Outcome evaluation is difficult for several reasons: co-existing extra-cranial injuries (spinal cord damage, for instance) may affect independence and quality of life outside the pure TBI effects; scales may not capture subtle, but important, changes; co-operation from patients may be impossible in the most severe cases. Several instruments have been developed for capturing specific aspects, from generic health status to specific cognitive functions. Even simple instruments, however, have demonstrated variable inter-rater agreement.

The possible links between structural traumatic brain damage and functional impairment have been explored both experimentally and in the clinical setting with advanced neuro-imaging techniques. We briefly report on some fundamental findings, which may also offer potential targets for future therapies.

Better understanding of damage mechanisms and new approaches to neuroprotection-restoration may offer better outcomes for the millions of survivors of TBI.

## Background

Traumatic brain injury (TBI) affects millions of people worldwide. Its incidence (new cases per year), including the whole range of severity from minor to devastating injuries, varies in different countries, from 60 cases per 100,000 inhabitants up to figures 12 times higher [1], reflecting local variations and, most likely, different inclusion criteria and methodologies. Epidemiology is changing, with more TBI due to increased motorization in several developing countries, while ageing of the population in the Western hemisphere increases the incidence of injuries due to falls in the elderly [2].

Mortality in severe TBI was higher than 50 % (up to 80 % in cases older than 60 years) in an old series [3]. Results have improved over the past decades and mortality has been reduced to 30–40 % [4]. The increased number of survivors, however, includes both successful cases who are back to an enjoyable life and cases with persistent disabilities (Table 1).

**Table 1:** Outcome at 6 months (percent data) from major pharmacological trials and consecutive series

	Publication year	Patient number	Death	Vegetative state	Severe disability	Moderate disability	Good recovery
Neuroprotective trials: placebo groups							
Tirilazad [82]	1998	459	28	4	13	17	38
Metilprednisolone [48]	2005	4819	22	Included in the mortality rate	14	17	46
Progesteron [83]	2014	588	22	Not reported	27	19	31
Consecutive series							
EBIC [84]	1999	796	31	2	16	20	31
NeuroLink [85]	2012	1273	33	3	14	17	33
UK RAIN study [86]	2013	2620	26	Not reported	33	22	19

Results at 6 months shown in this table are not corrected for severity. Better results in the neuroprotective trials may depend on the exclusion of the most severe cases, who are not amenable to randomization but are, on the contrary, included in consecutive series. The Metilprednisolone study included severe and moderate TBI

A proportion of severe TBI survivors, after prolonged hospital care, require long rehabilitation and may have long-term physical, cognitive, and psychological disorders. Such disorders may disrupt previous relationships and preclude return to work, with severe economic and social impacts. The global burden is such that TBI survivors have a lower life expectancy than the general population [4].

The weight of chronic consequences relative to medical care and rehabilitation costs has been estimated by calculating lifetime costs per case of severe TBI in the USA: 80 % of the estimated total cost (approximately USD 400,000) was attributable to disability and lost productivity [4].

The fact that severe brain damage is linked to harsh, long-lasting consequences is not unexpected. New data suggest, however, that disability may be common also after hospital admission for (apparently) mild head injuries. Accurate follow-up of 549 cases in Scotland estimated moderate or severe disability after mild TBI in 42–52 % of cases. A Canadian systematic review on the consequences of mild TBI, however, gives much lower figures [5]. Existing data are, therefore, insufficient to draw conclusions.

Figures on the prevalence of people living with the consequences of TBI are even less well documented; it has been estimated, however, that several million people (approximately five million in the USA and seven million in Europe) were living with TBI-related disability 10 years ago [6, 7].

No data are available for countries such as India and China, where TBI incidence is increasing due to motorization.

The consequences of injury may be attenuated by high quality care in the emergency setting, in the ICU, and over the whole rehabilitation process; family and social support also plays an important role. Economic and social disparities, with unequal access to resources and treatment, may, therefore, deeply influence outcome.

**Fig. 1:**
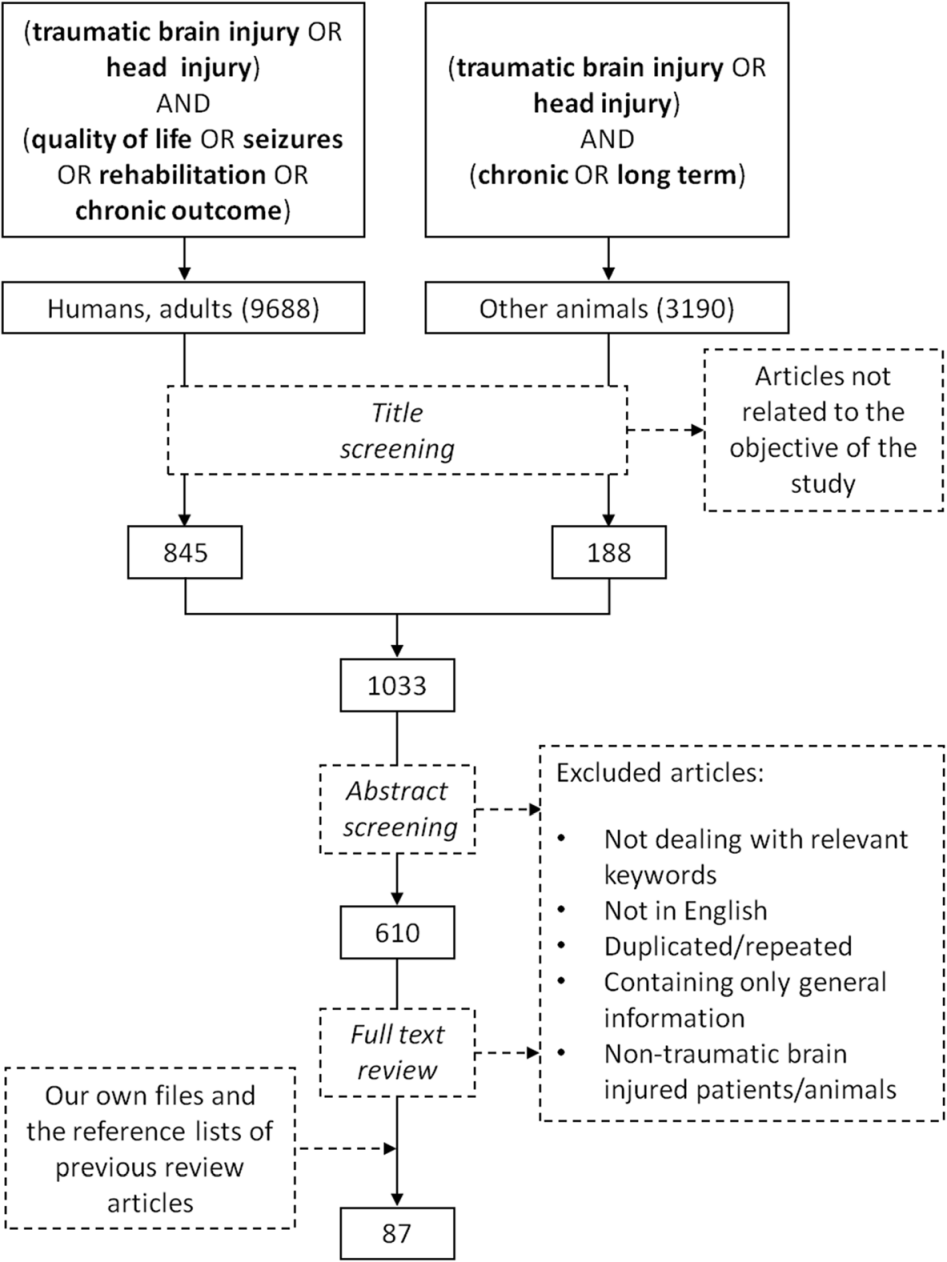
Flow diagram of literature search

This review is based on a comprehensive literature review which is detailed in Fig. 1. The selection process was conducted by the authors aiming at a narrative review, not a systematic literature review.

### Biology of traumatic brain injury

**Fig. 2:**
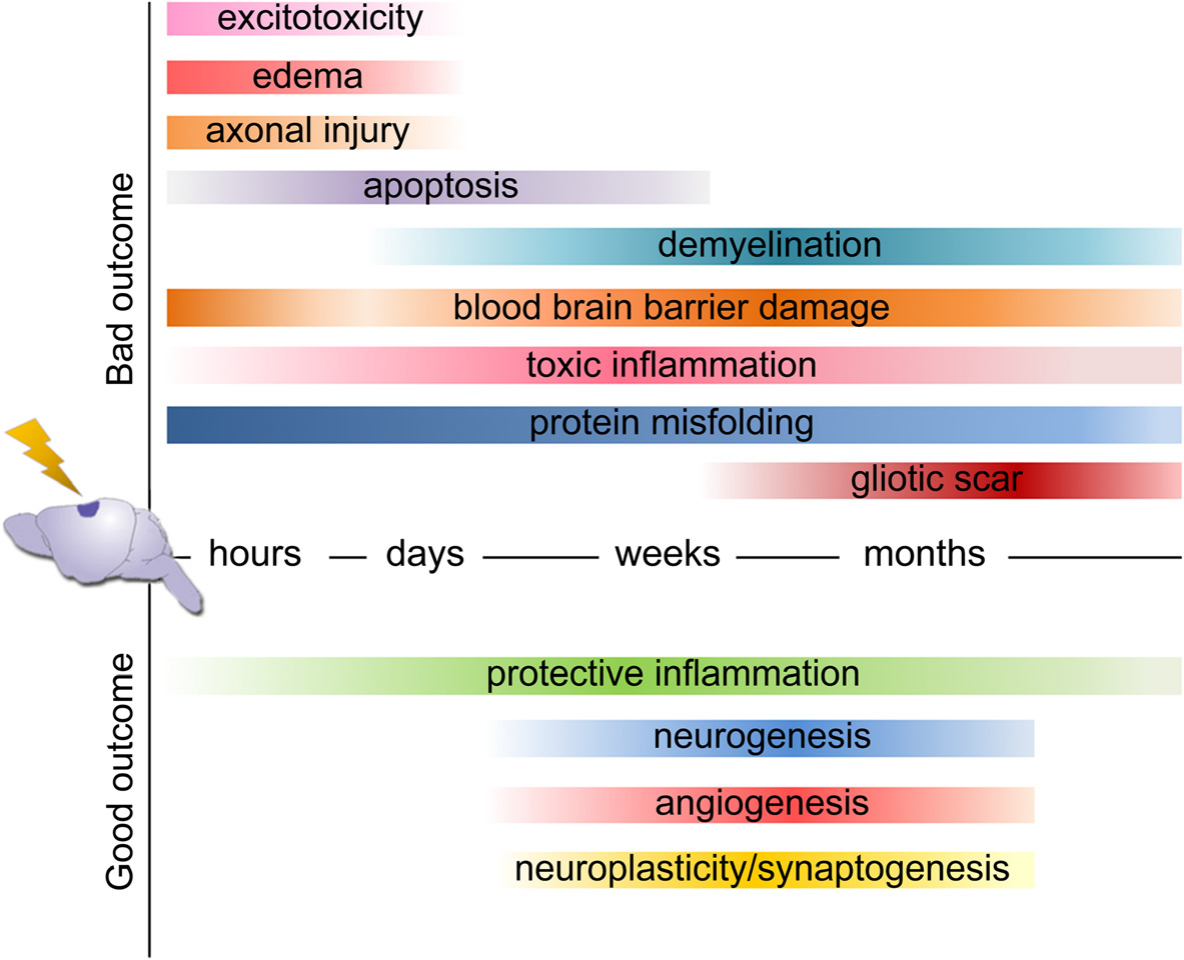
Toxic and protective events in TBI over time

Brain trauma is an acute biomechanical event characterized by multiple pathophysiological processes that develop over time in a continuum (Fig. 2). TBI survivors are affected by a “polypathology” whose main features are white matter degradation, neuronal loss, protein misfolding, and persistent neuroinflammation. Alterations of neurotransmitter systems have also been described [8].

#### White matter degradation

Neuronal circuits and functions depend on white matter integrity [9]. Seminal neuropathological studies by Adams and colleagues have documented the distribution of axonal pathology in a large series of TBI cases and have introduced the concept of “diffuse axonal injury” [10, 11]. Shear-tensile forces due to trauma may cause a disruption of the axonal cytoskeleton and impair axonal transport. Additional neurochemical changes, such as intracellular calcium overload, may further damage the axons. Thus, TBI affects structural brain networks progressively, from focal axon alteration to delayed axonal disconnection [12, 13].

**Fig. 3:**
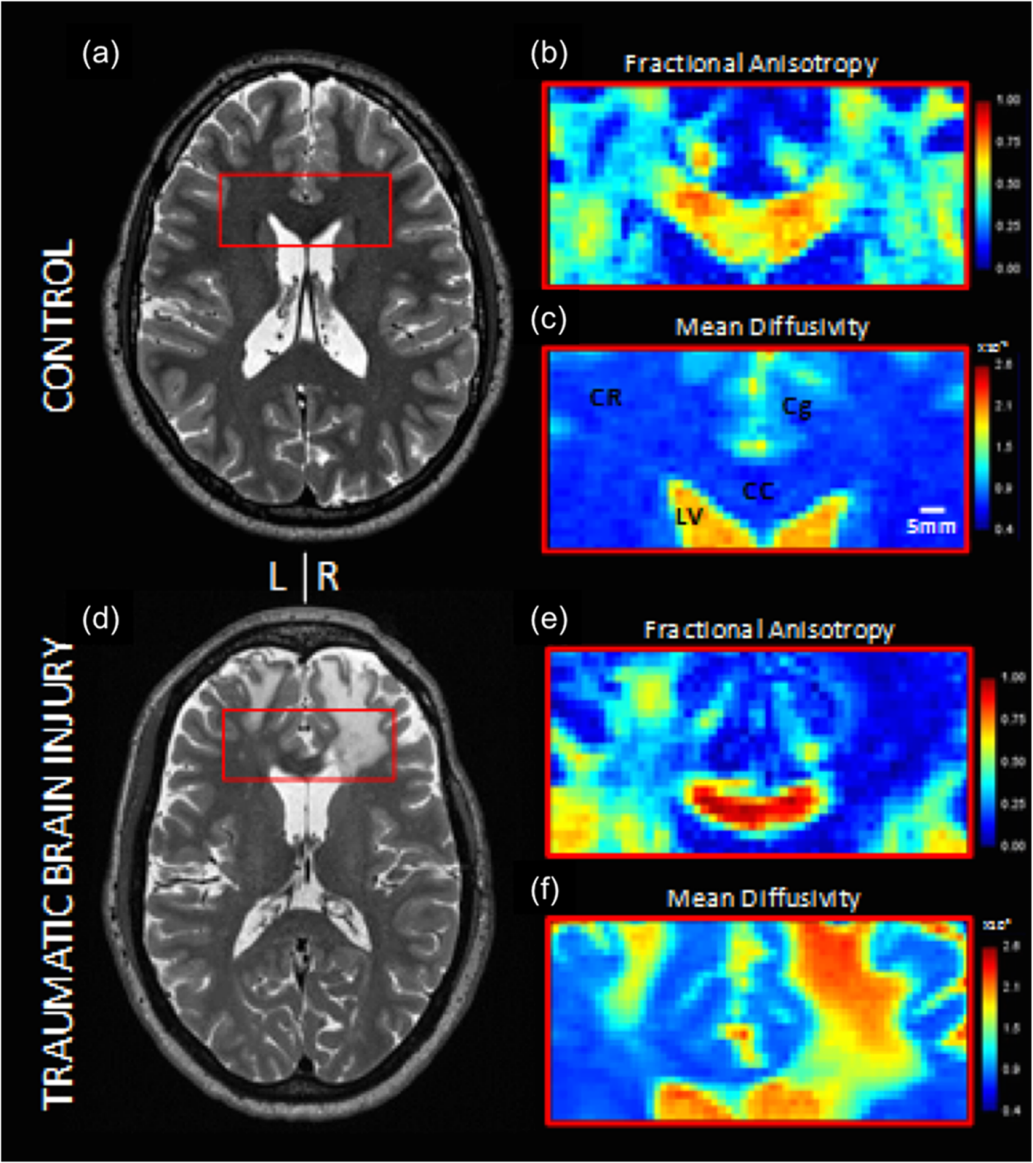
Advanced diffusion imaging in a normal control subject (**a**–**c**) and a TBI patient (**d**–**f**). **a**, **d** Axial T2-weighted images at 0.7-mm isotropic resolution. **b**, **e** Fractional anisotropy and **c**, **f** mean diffusivity from diffusion tensor imaging. *Cc* corpus callosum, *Cg* cingulum, *CR* corona radiata, *LV* lateral ventricle. The color scheme indicates quantitative diffusion parameters (not direction of fibers). Processing included averaging of two acquisitions with opposite phase encoding direction acquisitions and eddy current correction plus motion correction using the Human Connectome Project pipeline which included FSL 5.0.6. (L. Holleran, JH Kim, and DL Brody, unpublished data)

The functional and structural connectivity in patients can now be investigated by resting-state functional MRI and advanced diffusion imaging (Fig. 3), respectively, documenting axonal damage over a wide range of injury severities [8, 14, 15].

Studies indicate that even apparently intact axons with disrupted physiology may contribute greatly to clinical dysfunction in mild TBI. White matter abnormalities on advanced neuroimaging studies are evident in many patients in whom CT scans are normal and are strong predictors of long-term consequences [2, 9, 16, 17]. Serum markers of axonal injury are emerging (i.e., “SNTF”, a proteolytic fragment of alpha-II spectrin) that may assist in monitoring of neuropathology progression [18].

#### Protein misfolding

TBI is a risk factor for delayed neurodegeneration and dementia, including Alzheimer’s disease and chronic traumatic encephalopathy [19, 20]. Several mechanisms may be implicated, including axonal injury, neuronal loss, persistent inflammation, and prolonged blood–brain barrier disruption [21–24]. However, the neuropathological link that is receiving most attention is the accumulation of amyloid-β peptides and aberrant microtubule-associated protein tau, two common features in Alzheimer disease. Tau pathology has been shown to occur in rodent models of TBI within 2 weeks of injury [25–27]. Recent evidence indicates that a focal brain trauma in mice leads to persistent tau pathology which disrupts axonal microtubule networks, propagates to remote regions in the brain, and is associated with brain dysfunction [28].

#### Persistent inflammatory response

The inflammatory response in TBI includes local cerebral production of cytokines and chemokines, endothelial activation, microglial activation, and migration of systemic neutrophils, lymphocytes, and monocytes into the injured brain. In experimental TBI, microglial cells readily activate [29] and remain chronically activated for at least one year after injury [30], spreading form the site of injury to remote regions in the brain. Clinical data from TBI brain autopsies and from positron emission tomography of TBI patients identify chronic microglia activation up to several years after injury and document a close association between neuropathology and inflammation in space and time [31, 32]. Experimental studies show that aspecific suppression of the inflammatory response may protect the injured tissue early on but harm the brain at chronic stages [33], suggesting that therapeutic strategies should aim at modulation, rather than inhibition, of the inflammatory response.

#### Neurorestorative processes

In addition to toxic processes, TBI also induces neurorestorative events that include neurogenesis, gliogenesis, angiogenesis, synaptic plasticity, and axonal sprouting. These processes are stimulated by endogenous growth-related factors and may persist for weeks to months, contributing to recovery after TBI.

The adult brain retains neurogenic zones with neural stem cells that can differentiate into functional neurons [34, 35]. Several laboratories have reported an increased proliferative response in the hippocampus beginning as early as 2 days post-injury [36], with a peak in the first weeks after injury [37]. The proliferation in the dentate gyrus is age-dependent, with the juvenile brain showing a greater potential [36]. Newly generated neuroblasts [38] have been shown to migrate toward the site of injury [39] and to participate in cognitive recovery [40, 41]. Next to neurogenesis, axonal sprouting and synaptogenesis from surviving neurons may play a role in spontaneous motor recovery after TBI [42, 43]. However, all these spontaneous brain restorative processes are short-lived [44–46].

### Outcome assessment

Outcome assessment after TBI is complex and the specific consequences due to brain damage are sometimes difficult to identify. Brain trauma, especially in the case of road traffic accidents, is often associated with extra-cranial injuries. These injuries may themselves cause disabilities, sometimes to an overwhelming extent, as in case of concomitant spinal cord damage. Facial injuries, ocular damage, limb lesions or amputations, pelvic fractures, etc. are further examples of associated injuries potentially affecting quality of life (QoL) independent of, or in association with, brain damage.

Accordingly, pre-existing diseases may confuse the assessment: if TBI affects a person with a history of substance abuse or in the context of severe psychiatric disorders, the dissection of the pure effect of head injury becomes arduous or impossible.

The Glasgow Outcome Scale (GOS), a simple, five point scale, was designed in 1975 specifically for assessing outcome after TBI [47]. It became extremely popular as a simple tool to assess overall patient disability without detailed neurological and psychological evaluations, usually through a short interview. Its apparent simplicity was extremely attractive and the scale has been used in major clinical trials, such as CRASH [48], where approximately 10,000 cases were scored at 6 months after TBI. Limitations, however, were quickly identified: the broad categories couldn’t capture subtle changes, physical disabilities were better characterized than cognitive or behavioral problems, and the inter-rater agreement could vary widely [49].

Two major improvements have been subsequently introduced: an extended GOS scale, based on eight categories, and a structured interview for guiding the examiners toward a final accurate score [50]. Emphasis was put on assessing changes from the pre-injury status, accounting for pre-existing problems, and in assessing both mental and physical disabilities. The structured interview also suggested a simple exploration of social interactions, leisure attitudes, etc.

Despite the simplicity of the GOS and the guidance offered by the structured interview, discrepancies among raters remained a problem: when, during a trial on a neuroprotective agent, the results of interviews were reviewed centrally, more than one-third of the scores were discordant [51]. Alternative methods for reducing inter-rater differences using the GOS were then proposed [52], focusing on education and central monitoring.

Objective measurements of mental and physical function, as performed using a range of tools, may miss the patient’s own perspective on QoL, while he/she is the most qualified expert for evaluating the quality of his/her own life.

This could be captured by instruments for assessing the generic health status, such as the Medical Outcomes Survey 36-Item Short Form Health Questionnaire (SF-36) [53], or by tools specifically designed for TBI, such as the Quality of Life after Brain Injury (QOLIBRI), a 37-item scale with six subscales covering areas of wellbeing and functioning that are typically affected by TBI, plus a total score which provides a summary of QoL [54].

QoL is usually self-reported but self-reporting is not suitable for the most severe cases, with aphasia or persistent vegetative status.

Ideally a combination of instruments, such as the extended GOS and the QOLIBRI or other additional tools, could document important domains that are often not sufficiently investigated, such as interpersonal relationships, social and leisure activities, self and the environment, etc. [55]. Using complicated and long tests, however, may be cumbersome or unbearable for patients: when accurate testing was attempted in more than 1000 patients enrolled in the Traumatic Coma Data Bank, less than 100 completed the full test battery [56].

### Outcome changes over time

The temporal profile of outcome was first established in the first months following TBI, based on the observation that a significant proportion of patients improve continuously during the first 6 months after injury, stabilizing thereafter. When 786 TBI patients were followed up for 1 year, 35 % achieved a favorable outcome at 6 months and an additional 5 % reached this level at one year [57]. The finding that the most significant improvements, especially in the physical status, happen during the first 6 months has been confirmed in more recent series [58]. For this reason almost all major TBI trials in the past 20 years have assessed outcome 6 months after injury.

Evidence is accumulating, however, that outcomes after TBI may change after greater periods of time after trauma, in the direction of both further recovery and, unfortunately, progressive worsening. Additionally, some evidence suggests that TBI is a trigger of a persistent, chronic disease, with late deterioration several years after injury [19]. The evidence that about 25 % of TBI cases show functional deterioration between 7 and 13 years post-TBI indicates that, in some patients surviving the acute injury, TBI may be the substrate for the induction of neurodegenerative chronic processes.

### Long-term consequences

#### Excess mortality

TBI patients have a higher mortality rate than controls matched for age and sex. Behavioral problems, impulsivity, suicide, motor accidents, etc. are more common in young survivors, while in cases older than 45 years medical problems such as pneumonia, sepsis, and neurodegenerative diseases are associated with early deaths. In an American study the risk of dying was 2.2 times more than controls considering moderate to severe TBI who received inpatient rehabilitation, with an average reduction of life expectancy of 6.6 years [59]. The data were much worse for individuals who were unable to follow commands on admission to rehabilitation: they were 6.9 times more likely to die, with an average life expectancy reduction of 12.2 years.

#### Vegetative status and minimally conscious state

The vegetative state (or “unresponsive wakefulness syndrome”) is a complex neurological condition in which patients appear to be awake but show no sign of awareness of themselves or their environment [60]. This condition may be transient, preceding further recovery, or persist. If repeated accurate assessments confirm unresponsiveness 1 year after injury, a persistent vegetative state is diagnosed [59]. A high rate of misdiagnosis is reported because of the barriers to communication from the patient and the environment, so that patients with minimal, but present, responses (minimally conscious state) are confused with cases without responsiveness. These responses can be detected by complementing clinical evaluation with electrophysiology [61] and sophisticated imaging techniques [62].

#### Physical disabilities

Motor and sensory deficits may persist as a consequence of specific traumatic damage to the underlying nervous structures. In the most severe cases, additional damage due to prolonged immobilization during hospital care, such as peri-articular calcification, may worsen recovery. Bladder and sphincter control may be impaired. All these physical disabilities may cause significant handicap and limit the return to a normal and productive life.

#### Dementia

TBI has been identified as a risk factor for dementia but this topic is still debated. A large retrospective cohort (more than 50,000 mild, moderate, and severe TBI cases) identified 4361 (8.4 %) cases who developed dementia. In a stratified adjusted analysis, moderate to severe TBI was associated with increased risk of dementia across all ages, whereas mild TBI appeared to be a more important risk factor only in older cases (65 years or older) [63].

#### Endocrinopathies

Individual hormonal deficiencies after adult TBI are greatly variable in different reported studies. Chronic dysfunction of the pituitary axis is observed in approximately 35 % of individuals who sustain a moderate-to-severe TBI. The most common deficiency is that of growth hormone (GH), followed by gonadotropin, cortisol, and thyroid [64]. GH replacement provides clinically relevant, long-term QoL benefits in TBI patients with severe hypopituitarism [65]. When hormone deficits are not recognized and managed appropriately, they may profoundly affect both the results of the rehabilitative efforts and the final outcome of the subjects.

#### Cognitive impairment

TBI causes deficits of attention, memory, information processing speed, and executive functioning. High-level cognitive functions depend on well functioning distributed brain networks and on finely regulated neurotransmitter systems [8, 15], which may be disrupted by injury. When a group of moderate to severe TBI cases was extensively studied through comprehensive neuropsychological screening, deficits in sustained attention, paired associate learning, and reaction time have been clearly shown [66].

The relationship between TBI severity and neuropsychological deficits has been studied up to 10 years after injury [67]. Fifty per cent of mild cases recovered complete cognitive competency, while an additional 20 % required “some help”. On the other end of the spectrum, only 30 % of the more severe cases fully recovered [68]. More encouraging data on mild injuries recovery are reported in a recent systematic review [5].

White matter damage in definite locations, as demonstrated by advanced imaging techniques, seems to be associated with specific disorders: for instance, lesions to the fornices are correlated with associative learning and memory deficits; frontal lobe lesions are strictly linked with executive function impairment [15]. Grey matter lesions (especially in the orbitofrontal and insular cortices and in the caudate) seem associated with impulsivity; impairment of decision making, with longer deliberation times, also seems to be associated with a number of anatomical lesions [8].

As already mentioned, TBI is increasingly affecting an aging population in several countries; the neural loss that accompanies normal ageing might combine or interact with the brain damage caused by a TBI and worsen patients’ cognitive and social abilities [69, 70].

#### Psychiatric disorders

Psychiatric disorders are common following TBI and include depression, anxiety, and psychosis, as well as other maladaptive behaviors and personality changes. A recent meta-analysis shows that TBI increases the incidence of psychiatric disorders, with depression and bipolar disorders having higher odds ratios, 2.1 and 1.85, respectively. Psychiatric symptoms may be temporary, limited to the first weeks after injury, or persistent. They may limit participation in rehabilitation and functional independence in the community. Long-term psychiatric disorders are associated with greater risk for substance abuse [71].

#### Seizures

The incidence of seizures after TBI is variable, depending on the mechanism, the location, and the extent of brain damage and on appropriate treatment. Penetrating injuries are very often the cause of seizures, which may affect up to 50 % of patients. In closed TBI, the incidence of late seizures is lower but it may vary between 9 and 42 % in untreated patients [72]. Other sources indicate an incidence of 25–30 % after severe TBI and 5–10 % after mild to moderate injury [73]. There is low-quality evidence that early treatment with antiepileptic drugs reduces the risk of early post-traumatic seizures and no evidence to support a reduction in the risk of late seizures [74].

#### Employment

The combination of physical and functional deficits discussed translates into a high rate of un-employment in survivors of TBI. Patients recovering from severe TBI are sometimes offered a sheltered working environment, while return to previous work positions is rare. In a USA series 73 % of cases with mild initial injury return to previous jobs; this proportion falls to 49 % for severe patients [68]. Even patients of working age with apparently favorable outcomes have difficulties in restarting their jobs: in a group studied in Norway 10 years after injury, the rate of employment was 58 % [67].

#### Sexuality

Brain injury can directly and indirectly affect important aspects related to sexuality and sexual function. Physical (for instance pituitary dysfunction) and psychological components (such as depression) may both result in impaired sexual activities. When sexual function has been studied 1 year after TBI with self-reports and structured interviews, significant disturbances were detected [75]; 29 % of participants reported dissatisfaction with sexual functioning, with a greater percentage of men reporting dissatisfaction.

Sexual issues and sexual needs are rarely discussed and managed during the rehabilitation phase after TBI [76].

#### Impairment of social and leisure activities

The combination of physical, cognitive, and emotional impairments creates a major obstacle for re-entry into the community. Decreased social contact, depression, and loneliness combined with reduced financial resources, unemployment, and physical disabilities may severely disrupt previous social networks and make social and leisure activities impossible [77].

When several parameters (neuropsychological functioning, emotional status, functional status, employment, and perceived QoL) were assessed in 201 patients with moderate or severe TBI up to 3–5 years after injury, recovery to pre-injury levels ranged from 65 % of cases with regard to personal care to approximately 40 % with regard to cognitive competency, major activities, and leisure and recreation [68]. These figures were related to initial TBI severity.

Other series measuring QoL and comparing it with matched comparators confirm these findings: TBI cases experienced worse general health, elevated probabilities of depression, social isolation, and worse labor-force participation rates. The most affected areas were social function, emotions, and mental health [78]. Patients typically report “somewhat lower life satisfaction and affect” as a consequence of TBI [79]. QoL is consistently worse in older patients, as documented after the evacuation of subdural hematomas [80].

### What can be done to minimize long-term consequences and improve outcome

TBI consequences can be attenuated with appropriate and prolonged care. When professional help is linked with family assistance, the results may further improve. This, however, requires an organized system of care, financial resources, and a solid supportive network of “next of kin”.

For these reasons people with very limited financial resources and/or no familiar and social support are exposed to the worse TBI consequences. A typical demonstration of this concept has been documented in a cohort of homeless TBI patients in Scotland. The homeless have a 5.4 times increased risk of TBI compared with the normal population. After discharge back to their condition of homelessness, they had double the mortality rate compared with homeless cases not hospitalized [81].

A key issue in TBI care is the temporal progression of tissue damage, with long-lasting pathological cascades. Angiogenesis, neurogenesis, and brain plasticity, spontaneous regenerative mechanisms induced after acute brain injury, are too weak to counteract damage progression. If those mechanisms could be modulated and strengthened, new therapeutic possibilities could be explored.

## Conclusions

Traditionally seen as a sudden, brutal event with short-term consequences, TBI may cause persistent, sometimes life-long, consequences.

A huge amount of work has been invested in improving early TBI care, from rescue to emergency surgical interventions, prevention of secondary insults, acute treatment of intracranial hypertension in intensive care, etc. Data bases with ten thousands of patients have been assembled to better define diagnosis, management, and prognosis in the acute phase. In contrast, fewer data, usually on a very limited numbers of cases, are available on long-term outcomes. This is striking, because bringing patients with head injuries back to an enjoyable life should be the ultimate goal of any treatment.

Important long-term consequences have been identified not only after severe TBI but also in a relevant proportion of cases previously classified as moderate or mild.

Better understanding of the damage mechanisms and new approaches to neuroprotection-restoration may offer better outcomes for millions of survivors of TBI.

## Abbreviations

GH: growth hormone
GOS: Glasgow Outcome Scale
QoL: quality of life
QOLIBRI: Quality of Life after Brain Injury
TBI: traumatic brain injury
